# Development and Validation of a Bedside Score to Predict Early Death in Cancer of Unknown Primary Patients

**DOI:** 10.1371/journal.pone.0006483

**Published:** 2009-08-03

**Authors:** Nicolas Penel, Sylvie Negrier, Isabelle Ray-Coquard, Charles Ferte, Patrick Devos, Antoine Hollebecque, Michael B. Sawyer, Antoine Adenis, Pascal Seve

**Affiliations:** 1 Département de Cancérologie Générale, Centre Oscar Lambret, Lille, France; 2 Equipe d'Accueil 2694: Santé Publique, Epidémiologie et modélisation des maladies chroniques, Université de Lille II, Lille, France; 3 Département d'Oncologie Médicale, Centre Léon Bérard, Lyon, France; 4 Département de Cancérologie Digestive et Urologique, Centre Oscar Lambret, Lille, France; 5 Délégation à la Recherche Clinique, CHRU Lille, Lille, France; 6 Medical Oncology Department, Alberta University, Edmonton, Canada; 7 Département de Médecine Interne, Hôtel Dieu, Hospices Civiles de Lyon, Lyon, France; 8 INSERM U 590, Université de Lyon 1, Lyon, France; University Medical Center Rotterdam, Netherlands

## Abstract

**Background:**

We have investigated predictors of 90-day-mortality in a large cohort of non-specific cancer of unknown primary patients.

**Methods:**

Predictors have been identified by univariate and then logistic regression analysis in a single-center cohort comprising 429 patients (development cohort). We identified four predictors that produced a predictive score that has been applied to an independent multi-institutional cohort of 409 patients (validation cohort). The score was the sum of predictors for each patient (0 to 4).

**Results:**

The 90-day-mortality-rate was 33 and 26% in both cohorts. Multivariate analysis has identified 4 predictors for 90-day-mortality: performance status>1 (OR = 3.03, p = 0.001), at least one co-morbidity requiring treatment (OR = 2.68, p = 0.004), LDH>1.5×the upper limit of normal (OR = 2.88, p = 0.007) and low albumin or protein levels (OR = 3.05, p = 0.007). In the development cohort, 90-day-mortality-rates were 12.5%, 32% and 64% when the score was [0–1], 2 and [3]–[4], respectively. In the validation cohort, risks were 13%, 25% and 62% according to the same score values.

**Conclusions:**

We have validated a score that is easily calculated at the beside that estimates the 90-days mortality rate in non-specific CUP patients. This could be helpful to identify patients who would be better served with palliative care rather than aggressive chemotherapy.

## Introduction

Cancer of unknown primary (CUP) site represents about 2% of all invasive cancers diagnosed in adults (in 2006, 27,860 of 1,399,790 new cancer cases in the US) [1]. CUP is defined as a metastatic cancer with no identifiable origin at the time of diagnosis [2]. CUP is an aggressive cancer with generally poor outcomes; overall survival ranges from 4 to 12 months in large series [2]–[8]. Nevertheless, the recognition of particular clinico-pathologic entities and the specific treatments delivered to these patients significantly improved CUP management [8]. More recently, progress in immunochemistry [2]–[9] as well as gene profiling [10]–[11] made a step forward to better CUP diagnosis. However, these promising tools lack evidence in making impact on patient outcome and are of little use in daily practice.

But, 80% of CUP does not fall into favorable subsets [2]–[4]. Non-specific CUP treatment remains debatable, because its prognosis remains very difficult to estimate. Several previous studies have analyzed prognostic factors in such a population [4]–[7]. Nevertheless, these prognostic factors are not used in routine practice, because they are not convenient to use at the bedside [8]. From a physician's point of view it is of major importance to discriminate patients who would benefit from combination chemotherapy from those who would not and would be better served by palliative care.

Due to lack of reliable tools to estimate life-expectancy, we have conducted a new prognostic analysis in order to delineate and validate an easily derived bedside score that predicts risk of early death in CUP patients.

## Methods

### Development cohort

We retrospectively reviewed medical records of 429 consecutive patients primarily admitted to the Oscar Lambret Cancer Centre from November 1993 to February 2007. The study population consisted of patients who were diagnosed as having non-specific CUP. Inclusion criteria were: histological proof of malignancy, metastatic epithelial cancer, absence of identified primary site at the time of initial diagnostic and pre-treatment work-up. In addition, the following entities were excluded from analysis: adenocarcinoma in an axillary lymph node in women, primary papillary serous peritoneal carcinoma, undifferentiated carcinoma of the mediastinum and retroperitoneum in young men (middle line syndrome), cervical lymph nodes containing squamous cell carcinoma. All patients underwent a basic evaluation consisting on medical history, complete physical examination, biopsy and histopathological examination of the most easily accessible lesion, mammography for women, PSA levels for men, thoracic, abdominal and pelvic computed tomography (CT)-Scan, and, in the context of undifferentiated carcinoma the α-feto-protein and β-human chorionic gonadotrophin levels for both sexes [2].

### Validation cohort

This cohort included non-specific CUP referred to the Cross Cancer Institute, Edmonton, Canada from January 1998 to December 2004 (308 cases), to Centre Léon Bérard and Hospices Civils of Lyon, France from January 2000 to December 2004 (79 cases) and to Hospital of Lille University from January 2004 to November 2007 (22 cases) Lille, France.

### Primary endpoint

The primary endpoint was 90-day mortality. This threshold is believed to be relevant in decision-making for advanced cancer patients in whom the choice of whether to treat with chemotherapy or primary palliative care need to be discussed [12]–[15]

### Development of the score predicting the 90-day mortality

This analysis was conducted on the development cohort. We have first identified variables that predicted 90-day mortality using the Student t-test. Continuous variables were analyzed using Student t-test. Variables that predicted 90-day mortality were then dichotomized into binary variables using receiver-operator curves that estimated the cut-off optimizing both sensibility and specificity. Identifying predictors of 90-day-mortality among categorical variables was based on Chi-square tests and calculation of odds ratios and their 95%-confidence intervals (95%-CI).

Variables significantly associated with the 90-day-mortality in univariate analysis were then introduced into a stepwise logistic regression model [16]. Based on these analyses we developed a prognostic score. This score was calculated as the sum of predictors observed for each patient (from 0 to 4). Three categories of patients were defined: patients with high-risk of early death, patients with intermediate risk and patients with low risk according to observed death rates at each value of the score. Its performance was estimated using specificity (Se), specificity (Sp), positive predictive value (PPV), negative predictive value (NPV) and accuracy (rate of well classified) tabulated from a classical 2×2 table.

### Validating the model predicting 90-day mortality

This score was then applied to the validation dataset and its performance was estimated using the classical 2×2 table.

### Ethical Consideration

This study was conducted according to the principles expressed in the Declaration of Helsinki. The study was approved by the Institutional Review Board of the Oscar Lambret Cancer Center.

### Data processing and analyzing

The collected data were entered into computer and analyzed using SPSS version 13.0 statistical software.

The authors had also obtained the approval of Research Ethics Board of Alberta Cancer Board (ETH-21853, February 2006) and the approval of the French “Comission Nationale Informatique et Liberté” (date of approval June 2006)”.

## Results

### Study population

Development and validation cohorts are described in table 1. Median overall survivals were respectively 189 days (range 1–4,801) and 215 days range 1–3,842). The 90-day-mortality-rates were respectively 142/429 (33%) and 109/409 (26%).

**Table 1:** Patient's characteristics.

Categorical data		
Variables	Development dataset 429 cases (%)	Validation dataset 409 Cases (%)
Men	296 (68)	203 (49)
Women	133 (32)	206 (51)
PS = 0	141 (33)	57 (14)
PS = 1	138 (32)	129 (31)
PS = 2	108 (25)	103 (25)
PS = 3	35 (9)	93 (23)
PS = 4	4 (1)	26 (7)
Absence of co-morbidity or co- morbidity not requiring treatment	241 (56)	264 (66)
At least 1 co-morbidity requiring treatment	172 (44)	141 (34)
Number of met. site = 1	168 (39)	184 (45)
Number of met. site = 2	107 (25)	130 (31)
Number of met. site = 3	85 (20)	59 (14)
Number of met. site = 4	43 (10)	23 (7)
Number of met. site≥5	26 (6)	13 (3)
Adenocarcinoma	272 (63)	210 (51)
Undifferentiated carcinoma	58 (13)	138 (34)
Squamous cell carcinoma	77 (18)	24 (6)
Others	22 (6)	37 (9)
Lung met.	103 (24)	88 (21)
Liver met.	144 (33)	174 (42)
Bone met.	156 (36)	117 (28)
Brain met.	32 (8)	1 (0)
Continuous		
Variables (units)	Development dataset Median (range)	Validation dataset Median (range)
Age (years)	59 (22–91)	65 (19–92)
LDH (IU/l)	660 (57–10,084)	428 (86–7,538)
Alkaline phosphatase (IU/l)	280 (31–7,423)	Not done
Hemoglobin level (g/dl)	12.5 (6–17,3)	12.3 (6–18.2)
Platelets (U/mm3)	320,000 (7,000–830,000)	374,000 (10,000–736,000)
Lymphocytes (U/mm3)	1,300 (220–6,830)	1,250 (100–99,2000)
Variables	Development dataset 429 cases (%)	Validation dataset Cases (%)
Protein levels (g/l)	68 (49–92)	69 (42–87)
Albumin levels (g/l)	32 (14–51)	36 (19–49)

Abbreviations: PS = performance status, met. = metastasis, LDH = lactate dehydrogenase, ULN = upper limit of normal, IU: international unit, U:unit.

### Predictors for 90-day mortality

This analysis was conducted on the development cohort. Three continuous variables were not predictive for 90-day-mortality: age (p = 0.090), lymphocyte count (p = 0.2206) and platelet count (p = 0.7535). Five continuous variables were predictive of 90-day mortality and then were dichotomized into binary variables using the cut-off value that optimized both sensibility and specificity in ROC curves: number of metastatic sites with a cut-off fixed at>2 sites, LDH level with a cut-off fixed at>1.5 times the upper limit of normal (ULN), alkaline phosphatase levels with a cut-off fixed at>ULN, hemoglobin levels with a cut-off fixed at<12 g/dl, hypoproteinemia with a cut-off fixed at<70 g/l and hypoalbuminemia with a cut-off fixed at<35 g/l. In further analysis, patients with low protein or albumin levels have been combined into a single group.

Under univariate analysis, thirteen categorical variables were predictive for 90-day-mortality: Performance status (PS)>1, at least one co-morbidity requiring treatment, presence of lung, liver, bone, adrenal, brain or rare metastases, presence of more than 2 metastatic sites, LDH>1.5×ULN, alkaline phosphatase>ULN, hemoglobin less than 12 g/dl and low albumin or protein levels (Table 2). These variables were then introduced in a logistic regression model that identified 4 independent predictive factors for early death: PS>1, at least one co-morbidity requiring treatment, LDH>1.5×ULN and low protein or albumin levels.

**Table 2:** Identification of predictive factors for 90-day-mortality.

	Univariate analysis		Logistic regression	model
Variables not introduced in multivariate analysis	Odds Ratio and [95%-CI]	P value	-	-
Men	1.03 [0.60–1.76]	0.8204	-	-
Lymph nodes	0.68 [0.45–1.02]	0.063	-	-
Pleural met.	1.58 [0.78–3.18]	0.2006	-	-
Peritoneal met.	1.79 [0.89–3.60]	0.0980	-	-
Cutaneous met.	1.36 [0.38–4.89]	0.6398	-	-
Other histology than adenocarcinoma	1.03 [0.60–1.76]	0.3280	-	-
Variables introduced in multivariate analysis	Odds Ratio and [95%-CI]	P value	Adjusted Odds Ratio [95%-CI]	p
PS>1	4.70 [2.91–7.61]	<0.0001	3.03 [2.64–6.81]	0.0010
At least 1 co-morbidity requiring treatment	2.04 [1.29–3.23]	0.0015	2.68 [1.47–3.47]	0.0040
Lung met.	2.94 [1.80–4.83]	<0.0001	-	0.1580
Liver met.	2.59 [1.52–4.42]	0.0004	-	0.5640
Bone met.	1.47 [0.94–2.30]	0.0084	-	0.7000
Brain met.	2.61 [1.20–5.69]	0.0038	-	0.3300
Adrenal met.	4.34 [1.07–17.68]	0.0122	-	0.8890
Rare met.	2.42 [1.48–3.97]	0.0004	-	0.3430
Number of met. Site>2	2.94 [1.86–4.65]	0.0015	-	0.4400
LDH>1.5×ULN	3.18 [1.98–5.24]	<0.0001	2.88 [1.65–5.02]	0.0070
AP>ULN	2.01 [1.22–3.32]	<0.0001	-	0.8055
Hemoglobin<12 g/dl	2.67 [1.65–4.32]	<0.0001	-	0.3060
Low albumin or protein levels	3.93 [2.36–6.56]	<0.0001	3.05 [1.98–5.12]	0.0070

Abbreviations: 95%-CI: 95%-confidence intervals, PS = performance status, met. = metastasis, LDH = lactates dehydrogenase, AP = Alkaline phosphatase, ULN = upper limit of normal.

### Score and performance

**Figure 1:**
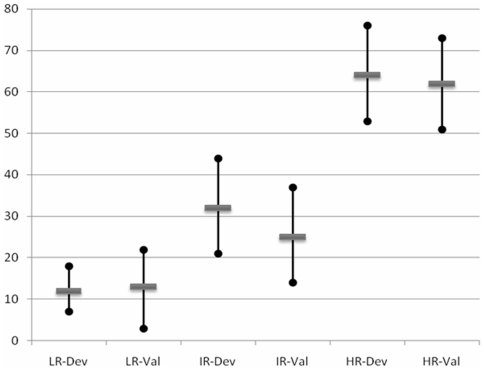
90-day-mortality-rates and 95%-confidence intervals according to the predicitve score. LR-Dev: Low-risk patients among the developpement cohort (score = [0–1]). LR-Val: Low-risk patients among the validation cohort (score = [0–1]). IR-Dev: Intermediate-risk patients among the developpement cohort (score = 2). IR-Val: Intermediate-risk patients among the validation cohort (score = 2). HR-Dev: High-risk patients among the developpement cohort (score = [3–4]). HR-Val: High-risk patients among the validation cohort (score = [3–4]).

In the development cohort, 274 patients were fully assessable for the four predictive factors and the primary endpoint. In order to develop a simple and bedside model, patients with score [0–1], 2 and [3]–[4] points were respectively considered at low risk, intermediate risk and high risk of 90-day mortality. Rates of 90-day-mortality were 12.5% for “low-risk patients”, 32% for “intermediate-risk patients” and 64% for “high-risk patients”. The 95%-confidence intervals (CI) of these three rates did not overlap (Table 3 and Figure 1). Performance of this score for prediction of 90-day-mortality were calculated in Table 3; accuracy and specificity were superior to 75% with a threshold set at score≥3 (that is to say when considering patients at high risk of 90 days mortality).

**Table 3:** Predictive score and its performance.

	Development cohort	Validation cohort
Assessable patients	274	174
90-day-mortality-rate	31% [25–36]	37% [30–44]
90-day-mortality-rates	according to the score	
Score = [0–1]		
Rate, 95%-CI	17/136 (12%), [7–18]	6/47 (13%), [3–22]
Score = 2		
Rate, 95%-CI	22/67 (32%), [21–44]	14/55 (25%), [14–37]
Score = [3–4]		
Rate, 95%-CI	46/71 (64%), [53–76]	45/72 (62%), [51–73]
Performance of the score	for prediction of 90-day- mortality	(Score≥3)
Sensitivity	0.54 [0.43–0.65]	0.69 [0.58–0.80]
Specificity	0.86 [0.82–0.91]	0.75 [0.67–0.83]
Positive predictive value	0.64 [0.53–0.75]	0.62 [0.51–0.73]
Negative predictive value	0.80 [0.75–0.86]	0.80 [0.72–0.88]
Accuracy	0.76 |0.68–0.82]	0.73 [0.66–0.79]

### Validation of the score

This score was then applied to the validation cohort. Only 174 patients were fully assessable for the four predictive factors and the primary endpoint. The separation of patients into the three groups was similar to that of the development cohort (Table 3 and Figure 1). In the validation cohort, 90-day-mortality-rates were 13%, 25% and 62% according to the score (Figure 1).

## Discussion

This retrospective analysis was conducted on a large database of patients with non-specific CUP. This study has generated an easily obtained at bedside score that estimate the risk of 90-day-mortality in such a population. Our multivariate analysis has identified four independent predictive factors: PS>1, presence of at least one underlying co-morbidity requiring treatment, elevated LDH and low albumin or protein levels. The 90-day-mortality rate in patients having at least 3 factors was about 62–64% (see Figure 1). This group of poor prognosis patients was well identified; the 95%-CI of the rate did not overlap the 95%-CI of other categories (see Figure 1). This is a reliable guidance to estimate the risk of early death and for rational decision making shared with patient.

Patient's characteristics were consistent with the literature on CUP patients. The 90-day mortality was 26% (120/350) in the Van der Gaast's series [5] and 33% (134/401) in the Culine's series [6]. Culine *et al.* has shown that LDH levels and PS constitute two major prognostic factors for CUP [6]. Van de Gaast *et al.* has also identified PS as major prognostic factor for CUP [5]. Seve *et al.* has previously shown that co-morbidity was also an important prognostic factor [17].

In the present study, LDH appears as one of the independent predictors for 90-day mortality. Although LDH is related to tumor burden, LDH is also high in liver diseases, in hemolysis and in other situations with massive cells destruction. Despite its lack of specificity, LDH remains a well-established prognostic factor for many metastatic diseases [6], [18]–[21].

Low albumin and protein levels are associated with both weight loss and induction of systemic inflammatory responses. These elements are interlinked in the metastatic setting, and hypoalbuminemia is a frequent biological sign of advanced disease. Serum albumin is a well-established marker of nutritional status and general patient status [18], [21], [23]–[24]. The prognostic value of this parameter is also well-established [12], [18], [21].

Despite its subjective nature, estimation of general condition by PS remains one of the most powerful prognostic factors in CUP patients [5]–[6]. Biological markers (LDH, albumin) that constitute more objective variables did not outperform PS in our model and in previously published ones [5]–[6].

As previously reported [17]–[18], co-morbidity requiring treatment constitutes the fourth predictor for 90-day mortality. This relationship we believe relates to our ability to treat the patient. It is noteworthy that in the present study and in previously published ones that age is not a prognostic factor in CUP patients. Nevertheless, severe underlying diseases limit our ability to administer optimally chemotherapy. Evaluation of co-morbidities could be done using the ACE-27 score; ACE-27>2 represented the cut-off used in the present study [17].

This study presents several limitations due to its retrospective nature. First of all, missing data did not allow analysis of the entire cohorts [7]. Extensive immunohistochemical analysis and gene profiling were not available. Several recent studies have shown the importance of molecular and histological expertise in this field, histological review of case must be discussed [25–27]. But despite these modern investigations, the vast majority of CUP remains without identifiable or highly-suspected underlying primary. Lastly, treatments were heterogeneous across study periods and study sites. Nevertheless, there is no consensus on treating non-specific CUP.

To conclude, we have developed and validated a score that is easily obtained at bedside that helps physicians to manage patients with non-specific CUP in a more rationale way. Further studies are required to combine this score with more current biological parameters (such as gene profiling). Use of large multi-institutional database could be useful to further narrow 95%-confidence intervals of each predictor and refine their roles in the final score. In a further analysis we plan to compare this score to the others published predictive tools [4]–[7]. A randomized trial comparing benefits of palliative chemotherapy versus best supportive care in patients having 3 or 4 predictors for early-death should be performed.
